# Extracellular binding of indinavir to matrix metalloproteinase-2 and the alpha-7-nicotinic acetylcholine receptor: implications for use in cancer treatment

**DOI:** 10.1016/j.heliyon.2019.e02526

**Published:** 2019-09-30

**Authors:** Anna Lee, Erin Saito, Sean Ekins, Aaron McMurtray

**Affiliations:** a Wayne State University School of Medicine, Detroit, MI, 48201, USA; b OC Neuroscience, Inc., Irvine, CA, 92604, USA; c Collaborations Pharmaceuticals, Inc., Raleigh, NC, 27606, USA

**Keywords:** Pharmaceutical science, Cancer research, Protease inhibitor, Alpha-7-nicotinic acetylcholine receptor, Matrix metalloproteinase-2, HIV-1 protease, Cancer, Indinavir

## Abstract

**Introduction:**

Results from recent studies have suggested a role for protease inhibitors in altering mechanisms involved in the initiation and proliferation of cancer cells. One such inhibitor, indinavir, may act as an anti-cancer agent by modulating the alpha-7-nicotinic acetylcholine receptor, which is a pro-carcinogenic protein that has been researched in conjunction with nicotine in lung cancer development. In our study, we compare indinavir's binding affinity towards α7-nAchR and MMP-2, another promoter of malignancy, to determine what extracellular effects the drug has before being internalized to inhibit HIV-1 protease.

**Methods:**

A computer program, PyRx, was used to compare indinavir's binding affinity with digital models for α7-nAchR, MMP-2 and HIV-1 protease, which were then compared to the results of *in vitro* binding assays for these targets.

**Results:**

PyRx testing predicted the highest binding affinity values for indinavir to MMP-2 (mean = 8.77 kcal/mol, S.D. = 0.29), followed by the α7-nAchR (mean = 8.53 kcal/mol, S.D. = 0.15) and HIV-1 protease (mean = 7.5 kcal/mol, S.D. = 0.44). *In vitro*, indinavir's mean percent inhibition of control values were 103.2 for HIV-1 protease, 5.3 for MMP-2, and 7.7 for the α7-nAchR.

**Conclusions:**

Binding affinity values for indinavir to MMP-2 and α7-nAchR were not significantly different. Using PyRx to predict affinity compared with in vitro testing did not yield comparable results. However, indinavir was shown to slightly inhibit both α7-nAchR and MMP-2, which may have ramifications in the drug's delivery to the intracellularly located HIV-1 protease.

## 1 Introduction

With an estimated incidence of 18.1 million cases worldwide in 2018 [1], cancer is a pressing, global issue; in the US alone, among adults between the ages of 40–79, cancer surpasses heart disease to hold the title as the leading cause of death [2], and the American Cancer Society estimates that in 2018, 1.7 million in the US will be newly diagnosed with cancer [3]. The implications of these numbers to the cost to society are paramount. Direct medical costs for cancer in the US were found to be $80.2 billion in 2015 [3], and with a growing population, these costs are projected to increase 34% from 2010 to 2020 [4]. In addition to its economic burden, cancer will claim around 609,640 US lives in 2018 [3], and the incidence of cancer will rise, though the rate of it is on the decline [5, 6]. Lung cancer, the leading cause of cancer deaths [2], has a 5-year survival rate of 56% when diagnosed early. However, only 16% of patients are diagnosed prior to metastasis, leading to an overall 5-year survival rate of 18% [3]. With such a bleak outlook, it is imperative that we improve our understanding, and ultimately, treatments for a disease that progressively continues to claim lives.

Cancer forms and spreads from its site of origin through a number of processes, such as cell cycle dysregulation [7] and epithelial-mesenchymal transition (EMT) [8, 9, 10, 11], respectively. Exposure to nicotine, an IARC group 1 carcinogen [12], leads to increased levels of VEGF, fibronectin, vimentin, Src, Raf-1 and other factors important in cell growth and invasion, and these downstream effects are initiated through the α7-nicotinic acetylcholine receptor (α7-nAchR) [13, 14]. In non-small cell lung cancer, the α7-nAchR is more highly expressed in smokers than nonsmokers [13], which provides a mechanism through which we can explain a poorer prognosis and increased metastasis for patients who smoke [15, 16, 17, 18, 19]. Antagonist compounds to α7-nAchR specifically, such as alpha-cobratoxin and alpha-bungarotoxin, have been able to inhibit cell growth *in vitro* and in mice [14], but additional compounds for safe, *in vivo* use in humans have yet to be discovered.

Several recent studies have shown promising results for HIV-protease inhibitors (PIs) in their anti-cancer properties. PIs, which are typically utilized in the treatment of HIV/AIDS, inhibit proteases that are necessary to cleave viral precursors into active proteins [20]. However, an alternative use for PIs in inhibiting invasion, angiogenesis, and in some cases, preventing proliferation of cancerous cells, has been established for several types of cancer [21, 22, 23]. More specifically, studies have indicated that some PIs inhibit matrix metalloproteinase-2 (MMP-2) [22,24]. Elevated MMP-2 levels have been correlated with metastasis [25] and a worse prognosis [26]. Other mechanisms of action, including endoplasmic reticulum (ER) stress [23], have been proposed for drugs like nelfinavir, indicating that these drugs also possibly work through apoptosis. Though reports are conflicting on whether some PIs affect cell proliferation, indinavir has been reported to affect MMP-2 and MMP-9, but not p21 [22], which emphasizes its importance in tumor metastasis, rather than initiation, specifically.

Recently, indinavir was identified to be a positive allosteric modulator (PAM) for the α7-nAchR when used at concentrations lower than 10 μM but an inhibitor when greater than 10 μM [27], potentially giving it additional anti-cancerous properties. Given this finding, we further explored indinavir's potential as an anti-cancer agent by evaluating its binding affinity towards α7-nAchR and MMP-2 with a computerized program, PyRx [28]. We also obtained *in vitro* data to compare it with the data from PyRx. Finally, we sought to compare these values to indinavir's binding affinity for HIV-1 protease, as the drug has an approved and established use on this protease in HIV treatment.

## 2 Methods

### 2.1 Computer modeling

For the selection of our compounds to be used in computer modeling, we used the 3D structures for the α7-nAchR and MMP-2 proteins from the National Protein Data Bank at www.rcsb.org (3SH1 and 1CK7, respectively) [29]; aplysia californica acetylcholine-binding protein (Ac-AchBP), mutated to reflect the binding characteristics of the human α7-nAchR [30], was used as a stand in structure for the receptor. The molecular structure for indinavir was downloaded from drugbank.ca.gov [31]. Using PyRx, a program available at http://pyrx.sourceforge.net [28], we minimized the energy of indinavir's compound and ran the program six times, as follows: three runs to obtain binding affinity values between indinavir and α7-nAchR, and another three runs to obtain binding affinity values between indinavir and MMP-2. Each run of the program listed different binding affinities between the two compounds that were obtained in the computer modeled analysis. We took the highest binding affinity value from each of the three runs between the two compounds, and obtained an average. We compared the average between indinavir and α7-nAchR and the average between indinavir and MMP-2 in our analysis.

Because HIV infection is the primary indication for indinavir usage, we compared indinavir's binding affinities for the two extracellular proteins versus indinavir's affinity for HIV-1 protease, an intracellular enzyme that indinavir competitively inhibits. The structure of HIV-1 protease (3HVP) was also downloaded from the National Protein Data Bank [29]. PyRx was then used to obtain binding affinity values between indinavir and HIV-1 protease. In a similar fashion to the data obtained for α7-nAchR and MMP-2, we ran 3HVP with indinavir three times, and also averaged the three highest values from each run.

### 2.2 In vitro testing

*In vitro* testing was accomplished through binding assays at Eurofins Cerep. Indinavir was tested at 10μM with α7-nAchR, HIV-1 protease and MMP-2 for percent inhibition of control values.

### 2.3 Data analysis

Mean and standard deviation values were calculated independently for the binding affinities between Indinavir and each of the protein targets (i.e., indinavir and α7-nAchR; indinavir and MMP-2; indinavir and HIV-1 protease). An analysis of variance (ANOVA) was used to determine if significant differences existed between the groups, using a p-value of less than 0.05 to indicate the presence of a significant difference. No correction for multiple comparisons was made, since there were only three groups. To identify differences in binding affinities between individual proteins, as well as between the extracellular (MMP-2 and α7-nAchR) and intracellular (HIV-1 protease) proteins, two-tailed t-tests were used, assuming unequal variances and using a p-value of less than 0.05 to indicate the presence of a significant difference. Normal distributions were assumed for all calculations. All calculations were performed using Microsoft Excel, version 15.24.

## 3 Results

The highest binding affinity values were found between indinavir and MMP-2 (1CK7), which were 9.1, 8.6, and 8.6 kcal/mol, (mean = 8.77 kcal/mol, S.D. = 0.29). The α7-nAchR (3SH1) bound to indinavir with 8.4, 8.7, and 8.5 kcal/mol, (mean = 8.53 kcal/mol, S.D. = 0.15). HIV-1 protease's (3HVP) binding affinity values to indinavir were 7.8, 7.7, and 7.0 kcal/mol, (mean = 7.5 kcal/mol, S.D. = 0.44).

The F-statistic value for ANOVA was 13.79, with p = 0.0057. This prompted us to run individual t-tests. Binding affinity values for MMP-2 and HIV-1 protease were significantly different, with p = 0.025. The p values for both α7-nAchR vs HIV-1 protease and for MMP-2 vs α7-nAchR were 0.061 and 0.304, respectively, suggesting no significant difference. Extracellular protein (both MMP-2 and α7-nAchR) binding affinity values collectively were also significantly different from intracellular (HIV-1 protease) values, with p = 0.024.

*In vitro*, indinavir's mean percent inhibition of control values were 103.2% for HIV-1 protease, 5.3% for MMP-2, and 7.7% for the α7-nAchR, suggesting the highest affinity between indinavir and HIV-1 protease *in vitro.*

## 4 Discussion

Cancer's grim outlook applies globally. By discovering the precise mechanism of its formation and spread, healthcare providers can tailor a patient's treatment to work more effectively and successfully. Chemotherapy and radiation broadly target replicating cells, and patients experience a myriad of debilitating side effects, such as nausea and vomiting, loss of appetite, and fatigue [32]. Conversely, indinavir and other HIV PIs have been discovered to potentially target specific pro-oncogenic agents, such as MMP-2 [22,24]. PIs inhibit invasion and some proliferation of cancerous cells, but the exact series of events through which it exerts its effects is unknown. Recently, indinavir was found to have characteristics that allow it to bind to the α7-nAchR [27], a receptor that plays a role in some types of cancer. We have now compared the binding affinities of two extracellular proteins, the α7-nAchR binding domain and MMP-2, to indinavir. Essentially, we are asking if the drug, prior to entering the cell, is more likely to work through its effects on MMP-2, α7-nAchR, or a combination of both.

Through PyRx, we found that differences between indinavir's affinity towards the extracellular proteins, MMP-2 and the α7-nAchR, were not statistically significant. *In vitro* testing of these two compounds also yielded similar results. Consequently, there may be different responses to indinavir in tissues where the concentration of these two extracellular proteins vary. For example, α7-nAchR expression is upregulated in smokers and in some malignant tissues, such squamous cell carcinoma lung cancer [13, 33]; the downstream effects of α7-nAchR activation lead to cell proliferation, especially in tissues where tumors were already formed [14]. In these cases, indinavir may be exerting more of its action as an inhibitor of the α7-nAchR, regardless of a similar binding affinity. In other tissues with similar MMP-2 and α7-nAchR concentrations, indinavir could have additive or synergistic anti-cancerous effects from interaction with both proteins. Further work would be necessary to determine the different clinical presentations and treatment outcomes among tissues of varying MMP-2 and α7-nAchR concentrations and to address any potential for synergy.

Alternatively, indinavir's affinity for these extracellular proteins may prove to be less beneficial outside the realm of cancer treatment. Indinavir is most commonly used in HIV/AIDS treatment, where it inhibits HIV-1 protease [34], an intracellular enzyme necessary for viral replication and survival [35]. In our analysis of computer model results, both extracellular proteins (MMP2- and α7-nAchR) collectively had significantly higher predicted binding affinities to indinavir than did HIV-1 protease. However, indinavir had a much higher percent inhibition with HIV-1 protease *in vitro*, though binding affinities, albeit lower, were still shown to exist for MMP-2 and α7-nAchR. The drug is characterized by a low plasma concentration and short time of action *in vivo*, and it requires frequent dosing (800 mg every 8 h) [36]. Because the latter two proteins are extracellular *in vivo*, there may still be significant ramifications on indinavir's delivery to HIV-1 protease, as it must enter the cell after encountering the two extracellular proteins. When treating HIV, a considerable amount of the drug may be complexed to either MMP-2 or α7-nAchR and unable to enter the cell to inhibit HIV-1 protease. Future studies could attempt to develop a structural analog of indinavir with a much lower or nonexistent binding affinity to MMP-2 and the α7-nAchR, which could overcome these hurdles and become more effective in reaching and acting on HIV-1 protease in HIV/AIDS affected individuals. High doses of indinavir, which is excreted by the kidney, may lead to nephrotoxicity [37, 38], and decreasing the dose has been associated with improved kidney function [39]. By allowing a larger proportion of the drug to enter the cell, lower doses could be administered, thereby potentially alleviating some of the negative side effects associated with higher doses of indinavir. However, nephrotoxicity still remains as a limitation for using indinavir in cancer treatment.

For patients with access to highly active antiretroviral therapy (HAART), including PIs, HIV is now a more manageable condition. However, almost half of virally suppressed HIV/AIDS patients using HAART medications experience mild cognitive dysfunction [40, 41], which raises the issue of the potential neurotoxicity of antiretroviral therapy [42]. A possible mechanism, through indinavir's inhibitory effects on the α7-nAchR, has been suggested, and this undesired consequence contradicts its beneficial response in cancer treatment. Therefore, further investigation would be required to determine a balanced dosage at which indinavir could work as a successful, anti-cancer agent without significant cognitive impairments; alternatively, concomitant medications, such as NMDA receptor antagonists, may be administered to offset this side-effect [27]. On the other hand, indinavir may be exerting advantageous effects through its actions on MMP-2. Studies have shown MMP-2 to be elevated in the central nervous system of patients with HIV related neurodegeneration [43, 44, 45], and by inhibiting MMP-2, indinavir may have a useful impact. This finding warrants further exploration of its effects *in vivo*.

Matrix metalloproteinase 9 (MMP-9) is another marker that has been widely referenced in studies of cancer, due to its ability to promote tumor invasion and metastasis. Higher levels of MMP-9 have been discovered in various types of cancer, which has led to its potential role as a biomarker in giant-cell tumor of the bone, non-small cell lung cancer and cervical cancer, among others [46]. Studies have shown lower levels of proteolytic activity for MMP-9 with use of indinavir, namely in cervical cancer [47, 48]. Additionally, indinavir decreased expression of MMP-9 from certain cell types [49]. These findings suggest that while indinavir's inhibition on MMP-9 has been well established, it may also have more of an indirect role in attenuating MMP-9's effects. In future studies, we believe that MMP-9, along with the two markers used in our study, MMP-2 and α7-nAchR, would be important targets in evaluating indinavir's potential as an anti-cancer agent.

Furthermore, PyRx and other similar programs may be useful in the development of pharmaceutical drugs. A recent study estimated a single drug's research and development costs to be $2.8 billion [50], and the combination of computational and *in vitro* approaches may improve the efficiency of drug discovery. This could not only lower R&D expenditures, but also avoid some undesired and serious side effects. However, it is worth noting that our PyRx results did not align with our *in vitro* testing, and thus may be limited in its practical use.

Though our PyRx results did not align with our *in vitro* testing, our study may have been limited in that the proteins we selected from the National Protein Data Bank may not have been the most representative model of its *in vivo* counterpart. Our study was also limited to just a few proteins in the extracellular space, as we only investigated MMP-2 and the α7-nAchR binding regions. Future efforts in determining indinavir's potential in cancer treatment should include testing of cancer cell lines, as well as including a wider range of concentration for *in vitro* testing of indinavir, rather than the 10 μM used in our study. Additionally, indinavir was selected as our protease inhibitor of choice for the study, based on previous data that showed its potential to inhibit the α7-nAchR [27]. Subsequent studies should evaluate other protease inhibitors' capabilities as anti-cancer agents.

## 5 Conclusion

In conclusion, our study explored using the PI, indinavir, against two potential extracellular binding targets (MMP-2 and α7-nAchR) for use in cancer treatment, and measured their binding values against that of HIV-1 protease, an intracellular target for indinavir in HIV/AIDS treatment. We compared the predicted binding and experimental inhibition from computational and *in vitro* data, respectively, and discussed how the method we used could benefit large-scale drug development projects.

## Declarations

### Author contribution statement

Anna Lee, Aaron McMurtray: Conceived and designed the experiments; Analyzed and interpreted the data; Wrote the paper.

Erin Saito, Sean Ekins: Conceived and designed the experiments; Wrote the paper.

### Funding statement

This work was supported by the Joseph Stahlberg Foundation.

### Competing interest statement

The authors declare no conflict of interest.

### Additional information

No additional information is available for this paper.

## References

[1] Bray F., Ferlay J., Soerjomataram I., Siegel R.L., Torre L.A., Jemal A. (2018). Global cancer statistics 2018: GLOBOCAN estimates of incidence and mortality worldwide for 36 cancers in 185 countries. CA Cancer J. Clin..

[2] Siegel R.L., Miller K.D., Jemal A. (2017). Cancer statistics, 2017. CA Cancer J. Clin..

[3] American Cancer Society (2018). Cancer Facts & Figures 2018. https://www.cancer.org/content/dam/cancer-org/research/cancer-facts-and-statistics/annual-cancer-facts-and-figures/2018/cancer-facts-and-figures-2018.pdf.

[4] Trogdon J.G., Tangka F.K., Ekwueme D.U., Guy G.P., Nwaise I., Orenstein D. (2012). State-level projections of cancer-related medical care costs: 2010 to 2020. Am. J. Manag. Care.

[5] Smith B.D., Smith G.L., Hurria A., Hortobagyi G.N., Buchholz T.A. (2009). Future of cancer incidence in the United States: burdens upon an aging, changing nation. J. Clin. Oncol..

[6] Edwards B.K., Howe H.L., Ries L.A., Thun M.J., Rosenberg H.M., Yancik R., Wingo P.A., Jemal A., Feigal E.G. (2002). Annual report to the nation on the status of cancer, 1973-1999, featuring implications of age and aging on U.S. cancer burden. Cancer.

[7] Hanahan D., Weinberg R. (2000). The hallmarks of cancer. Cell.

[8] Kang Y., Massagué J. (2004). Epithelial-mesenchymal transitions: twist in development and metastasis. Cell.

[9] Yang J., Mani S.A., Donaher J.L., Ramaswamy S., Itzykson R.A., Come C., Savagner P., Gitelman I., Richardson A., Weinberg R.A. (2004). Twist, a master regulator of morphogenesis, plays an essential role in tumor metastasis. Cell.

[10] Thiery J.P. (2002). Epithelial-mesenchymal transitions in tumour progression. Nat. Rev. Cancer.

[11] Huber M.A., Kraut N., Beug H. (2005). Molecular requirements for epithelial-mesenchymal transition during tumor progression. Curr. Opin. Cell Biol..

[12] International Agency for Research on Cancer, IARC list of classifications, volumes 1–120. http://monographs.iarc.fr/ENG/Classification/latest_classif.php (accessed July 2016).

[13] Zhao Q., Gu X., Zhang C., Lu Q., Chen H., Xu L. (2015). Blocking M2 muscarinic receptor signaling inhibits tumor growth and reverses epithelial-mesenchymal transition (EMT) in non-small cell lung cancer (NSCLC). Cancer Biol. Ther..

[14] Dasgupta P., Rizwani W., Pillai S., Kinkade R., Kovacs M., Rastogi S., Banerjee S., Carless M., Kim E., Coppola D., Haura E., Chellappan (2009). Nicotine induces cell proliferation, invasion and epithelial-mesenchymal transition in a variety of human cancer cell lines. Int. J. Cancer.

[15] Richardson G.E., Tucker M.A., Venzon D.J., Linnoila R.I., Phelps R., Phares J.C., Edison M., Ihde D.C., Johnson B.E. (1993). Smoking cessation after successful treatment of small-cell lung cancer is associated with fewer smoking-related second primary cancers. Ann. Intern. Med..

[16] Abrams J.A., Lee P.C., Port J.L., Altorki N.K., Neugut A.I. (2008). Cigarette smoking and risk of lung metastasis from esophageal cancer. Cancer Epidemiol. Biomark. Prev..

[17] Yu G.P., Ostroff J.S., Zhang Z.F., Tang J., Schantz S.P. (1997). Smoking history and cancer patient survival: a hospital cancer registry study. Cancer Detect. Prev..

[18] Murin S., Inciardi J. (2001). Cigarette smoking and the risk of pulmonary metastasis from breast cancer. Chest.

[19] Kobrinsky N.L., Klug M.G., Hokanson P.J., Sjolander D.E., Burd L. (2003). Impact of smoking on cancer stage at diagnosis. J. Clin. Oncol..

[20] Richman D. (2001). HIV chemotherapy. Nature.

[21] Esposito V., Palescandolo E., Spugnini E.P., Montesarchio V., De Luca A., Cardillo I., Cortese G., Baldi A., Chirianni A. (2006). Evaluation of antitumoral properties of the protease inhibitor indinavir in a murine model of hepatocarcinoma. Clin. Cancer Res..

[22] Toschi E., Sgadari C., Malavasi L., Bacigalupo I., Chiozzini C., Carlei D., Compagnoni D., Bellino S., Bugarini R., Falchi M., Palladino C., Leone P., Barillari G., Monini P., Ensoli B. (2011). Human immunodeficiency virus protease inhibitors reduce the growth of human tumors via a proteasome-independent block of angiogenesis and matrix metalloproteinases. Int. J. Cancer.

[23] Koltai T. (2015). Nelfinavir and other protease inhibitors in cancer: mechanisms involved in anticancer activity. F1000Res.

[24] Sgadari C., Barillari G., Toschi E., Carlei D., Bacigalupo I., Baccarini S., Palladino C., Leone P., Bugarini R., Malavasi L., Cafaro A., Falchi M., Valdembri D., Rezza G., Bussolino F., Monini P., Ensoli B. (2002). HIV protease inhibitors are potent anti-angiogenic molecules and promote regression of Kaposi sarcoma. Nat. Med..

[25] Cathcart J., Pulkoski-Gross A., Cao J. (2015). Targeting matrix metalloproteinases in cancer: bringing new life to old ideas. Genes Dis..

[26] Hadler-Olsen E., Winberg J.O., Uhlin-Hansen L. (2013). Matrix metalloproteinases in cancer: their value as diagnostic and prognostic markers and therapeutic targets. Tumour Biol..

[27] Ekins S., Mathews P., Saito E.K., Diaz N., Naylor D., Chung J., McMurtray A.M. (2017). α7-Nicotinic acetylcholine receptor inhibition by indinavir: implications for cognitive dysfunction in treated HIV disease. AIDS.

[28] Dallakyan S., Olson A.J. (2015). Pyrx. Small-molecule library screening by docking with PyRx. Methods Mol. Biol..

[29] Berman H.M., Westbrook J., Feng Z., Gilliland G., Bhat T.N., Weissig H., Shindyalov I.N., Bourne P.E. (2000). The protein Data Bank. Nucleic Acids Res..

[30] Nemecz A., Taylor P. (2011). Creating an α7 nicotinic acetylcholine recognition domain from the acetylcholine-binding protein crystallographic and ligand selectivity analyses. J. Biol. Chem..

[31] Law V., Knox C., Djoumbou Y., Jewison T., Guo A.C., Liu Y., Maciejewski A., Arndt D., Wilson M., Neveu V., Tang A., Gabriel G., Ly C., Adamjee S., Dame Z.T., Han B., Zhou Y., Wishart D.S. (2014). DrugBank 4.0: shedding new light on drug metabolism. Nucleic Acids Res..

[32] NIH National Cancer Institute (2018). Side Effects of Cancer Treatment. https://www.cancer.gov/about-cancer/treatment/side-effects.

[33] Brown K.C., Perry H.E., Lau J.K., Jones D.V., Pulliam J.F., Thornhill B.A., Crabtree C.M., Luo H., Chen Y.C., Dasgupta P. (2013). Nicotine induces the up-regulation of the α7-nicotinic receptor (α7-nAChR) in human squamous cell lung cancer cells via the Sp1/GATA protein pathway. J. Biol. Chem..

[34] US Department of Health and Human Services, AIDS Info. https://aidsinfo.nih.gov/drugs/233/indinavir/30/professional (accessed July 2016).

[35] Zhang S., Kaplan A.H., Tropsha A. (2008). HIV-1 protease function and structure studies with the simplicial neighborhood analysis of protein packing (SNAPP) method. Proteins.

[36] Lv Z., Yu C., Wang Y. (2015). HIV protease inhibitors: a review of molecular selectivity and toxicity. HIV AIDS (Auckl).

[37] Kalyesubula R., Perazella M.A. (2011). Nephrotoxicity of HAART. AIDS Res. Treat..

[38] Izzedine H., Launay-Vacher V., Deray G. (2005). Antiviral drug-induced nephrotoxicity. Am. J. Kidney Dis..

[39] Boyd M.A., Siangphoe U., Ruxrungtham K., Reiss P., Mahanontharit A., Lange J.M., Phanuphak P., Cooper D.A., Burger D.M. (2006). The use of pharmacokinetically guided indinavir dose reductions in the management of indinavir-associated renal toxicity. J. Antimicrob. Chemother..

[40] Robertson K.R., Smurzynski M., Parsons T.D., Wu K., Bosch R.J., Wu J., McArthur J.C., Collier A.C., Evans S.R., Ellis R.J. (2007). The prevalence and incidence of neurocognitive impairment in the HAART era. AIDS.

[41] Heaton R.K., Franklin D.R., Ellis R.J., McCutchan J.A., Letendre S.L., Leblanc S., Corkran S.H., Duarte N.A., Clifford D.B., Woods S.P., Collier A.C., Marra C.M., Morgello S., Mindt M.R., Taylor M.J., Marcotte T.D., Atkinson J.H., Wolfson T., Gelman B.B., McArthur J.C., Simpson D.M., Abramson I., Gamst A., Fennema-Notestine C., Jernigan T.L., Wong J., Grant I., CHARTER Group, HNRC Group (2011). HIV-associated neurocognitive disorders before and during the era of combination antiretroviral therapy: differences in rates, nature, and predictors. J. Neurovirol..

[42] Underwood J., Robertson K.R., Winston A. (2015). Could antiretroviral neurotoxicity play a role in the pathogenesis of cognitive impairment in treated HIV disease?. AIDS.

[43] Conant K., McArthur J.C., Griffin D.E., Sjulson L., Wahl L.M., Irani D.N. (1999). Cerebrospinal fluid levels of MMP-2, 7, and 9 are elevated in association with human immunodeficiency virus dementia. Ann. Neurol..

[44] Zhang K., McQuibban G.A., Silva C., Butler G.S., Johnston J.B., Holden J., Clark-Lewis I., Overall C.M., Power C. (2003). HIV-induced metalloproteinase processing of the chemokine stromal cell derived factor-1 causes neurodegeneration. Nat. Neurosci..

[45] Li S., Wu Y., Keating S.M., Du H., Sammet C.L., Zadikoff C., Mahadevia R., Epstein L.G., Ragin A.B. (2013). Matrix metalloproteinase levels in early HIV infection and relation to in vivo brain status. J. Neurovirol..

[46] Huang H. (2018). Matrix metalloproteinase-9 (MMP-9) as a cancer biomarker and MMP-9 biosensors: recent advances. Sensors (Basel).

[47] Barillari G., Iovane A., Bacigalupo I., Palladino C., Bellino S., Leone P., Monini P., Ensoli B. (2012). Ritonavir or saquinavir impairs the invasion of cervical intraepithelial neoplasia cells via a reduction of MMP expression and activity. AIDS.

[48] Sharma S., Baksi R., Agarwal M. (2016). Repositioning of anti-viral drugs as therapy for cervical cancer. Pharmacol. Rep..

[49] Liuzzi G.M., Mastroianni C.M., Latronico T., Mengoni F., Fasano A., Lichtner M., Vullo V., Riccio P. (2004). Anti-HIV drugs decrease the expression of matrix metalloproteinases in astrocytes and microglia. Brain.

[50] DiMasi J.A., Grabowski H.G., Hansen R.W. (2016). Innovation in the pharmaceutical industry: new estimates of R&D costs. J. Health Econ..

